# Anaplastic lymphoma kinase-negative pulmonary inflammatory myofibroblastic tumor with multiple metastases and its treatment by Apatinib: A case report

**DOI:** 10.1097/MD.0000000000018414

**Published:** 2019-12-27

**Authors:** Qiuxia Liu, Jianguo Wei, Xizhong Liu, Jianfang Wang

**Affiliations:** a Department of Medical Oncology; b Department of Pathology, Shaoxing People's Hospital, Shaoxing Hospital of Zhejiang University, Zhejiang; c Institute of Nonlinear Science, Shaoxing University, Shaoxing, China.

**Keywords:** apatinib, celecoxib, metastatic IMT, pulmonary IMT

## Abstract

**Rationale:**

Primary pulmonary inflammatory myofibroblastic tumor (IMT) with distant metastasis is extremely rare. Moreover, metastasis of pulmonary IMT to bone marrow has never been reported in previous studies. Therapeutic approaches for anaplastic lymphoma kinase (ALK)-negative pulmonary IMT with metastasis are limited. Yet there is no report on the treatment of advanced IMT cases with anti-angiogenesis drugs.

**Patient concerns:**

We described a patient with a complaint of fatigue, with the chest computed tomography (CT) scan revealing 2 masses in bilateral lung.

**Diagnoses:**

The CT-guided lung biopsy examined 1 lesion in the right lung, and the post-operative pathological diagnosis of ALK-negative pulmonary IMT was recommended. However, the lung lesions were found significantly enlarged during the subsequent visit 8 months later, along with multiple metastases to the bone and abdominal cavity. A bone marrow biopsy revealed bone marrow infiltration by spindle cells.

**Interventions:**

The patient began to take Celecoxib due to the rapid progression of IMT, however, resulting in the aggravated gastric ulcer. He stopped taking the medicine 1 month later, with no remarkable change in the lesions by CT. Apatinib was administrated instead of Celecoxib.

**Outcomes:**

After the 5-month treatment of Apatinib, the mass in the abdominal cavity significantly shrank and the lung lesions slightly decreased in size. With the 9-month administration of Apatinib, the lung lesions and the abdominal mass kept stable, compared with the situation in the 5-month follow-up.

**Lessons:**

Although pulmonary IMT shows the potential of metastasis, its metastasizing to bone marrow is a highly unusual event. Apatinib is effective for pulmonary IMT, and should be taken into consideration for the treatment of inoperable pulmonary IMT patients who lack ALK rearrangement.

## 1 Introduction

Inflammatory myofibroblastic tumor (IMT) is a rare neoplasm histopathologically composed of differentiated myofibroblastic spindle cells accompanied by inflammatory cells.^[1]^ It occurs in various anatomical locations, among which lung is the most common site followed by abdomen and retroperitoneum. IMT is generally more common in children and young people, accounting for <1% of all lung tumors. Although IMT is defined as an intermediate tumor with the potential for local recurrence or metastasis, distant metastasis—most likely occurring in extrapulmonary tumors—is seldom seen.^[2]^ Surgery is the most fundamental and effective treatment. Rearrangements involving the anaplastic lymphoma kinase (ALK) locus on chromosome 2p23 have been discovered in 50% of the cases with IMT.[^3^,^4^] As an ALK inhibitor, Crizotinib has been proved effective for ALK-rearranged patients. However, there is still no standard treatment for the patients with inoperable and ALK-negative IMT.

Pulmonary IMT with metastasis is extremely rare, which has been depicted only in sporadic case reports. Here, we report a case of IMT featuring primary lesions in bilateral lung with multiple metastatic tumors in the bone, abdominal cavity, and bone marrow. To our knowledge, this is the first case of the pulmonary IMT with metastasis to bone marrow. The treatment of Celecoxib followed by Apatinib, a tyrosine kinase inhibitor of vascular endothelial growth factor receptor-2 (VEGFR-2), was carried out in this patient, and the efficacy was evaluated. Ethics approval was gained from the Shaoxing People's Hospital Ethics Committee, and the patient consent was also obtained for the publication of this case report with the accompanying images.

## 2 Case presentation

**Figure 1:**
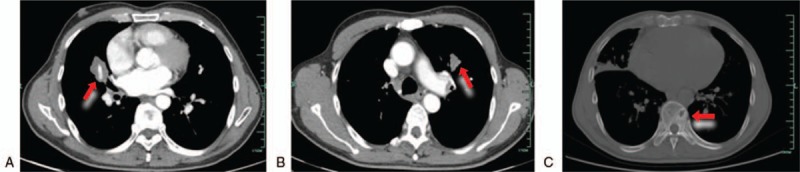
Chest computed tomoraphy scan showed a mass on the medium lobe of right lung (A), anther mass on the upper lobe of left lung (B), and thoracic vertebra metastases (C).

A 59-year-old man was admitted to our center with the chief complaint of consistent fatigue in March 2017. He had a 30-year history of hypertrophic obstructive cardiomyopathy, a 20-year history of chronic viral hepatitis B, and had suffered from gastric ulcers for five years. In addition, he often got pneumonia at least 2 or 3 times a year in the past few years. A Chest CT revealed a 3.7 cm × 2.1 cm mass encircling the main pulmonary artery in the right middle lobe of the lung and another 2.2 cm × 1.5 cm mass in the left upper lobe of the lung, both of which showed slightly contrast enhancements with clear boundaries (Fig. 1). However, the patient declined our suggestion for further examinations.

**Figure 2:**
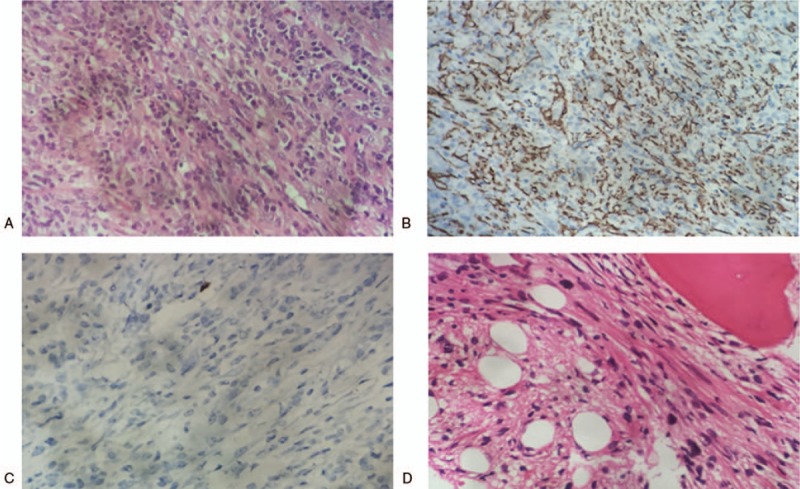
(A) Histological examination demonstrated that lung lesion was composed of spindle cells in the background of plasma cells and lymphocytes (hematoxylin and eosin stain, ×400). (B) Immunohistochemistry analysis showed that the spindle cells were positive for smooth muscle actin (×200). (C) Immunohistochemistry analysis showed that the spindle cells were negative for anaplastic lymphoma kinase 1 (×400). (D) Bone marrow was infiltrated by spindle cells (hematoxylin and eosin stain, ×400).

As fatigue aggravated 8 months later, he was admitted to our hospital for a second visit. A repeated CT showed that the mass of the right lung increased to 4.7 cm × 3.4 cm, with a little decrease in the lesion on the left side. Meanwhile, hepatic multiple enhanced nodular signals were also detected by the abdominal CT, but liver tumors were further excluded by the abdominal magnetic resonance image (MRI). Apart from fatigue, the patient did not have any other complaint, without cough, chest pain, fever, or other findings by physical examinations. The laboratory findings were negative, white blood cell count, hemoglobin level, platelet count, C-reactive protein, elevated sedimentation rate, and cancer-related antigen were within the normal ranges. The CT-guided lung biopsy of the mass in the right middle lobe of the lung was performed in December 2017. The corresponding histological results revealed that the mass comprised spindle cells without the infiltration of atypia inflammatory cells. The immunohistochemical examination showed that the cells were positive for smooth muscle actin (SMA) and Ki-67 (10%), but negative for CD34, S-100 protein, epithelial membrane antigen, and ALK_1_ (Fig. 2). Thus, the diagnosis of pulmonary IMT was rendered. Because of the anatomic complexity and the high anesthetic risk of hypertrophic obstructive cardiomyopathy, surgical resection was not appropriate for the patient. Considering the indolent behavior of this entity and his fear of side effects on gastric ulcer, the patient denied the treatments of steroids and nonsteroidal anti-inflammatory agents (NSAIDs). Therefore, a close follow-up was suggested.

**Figure 3:**
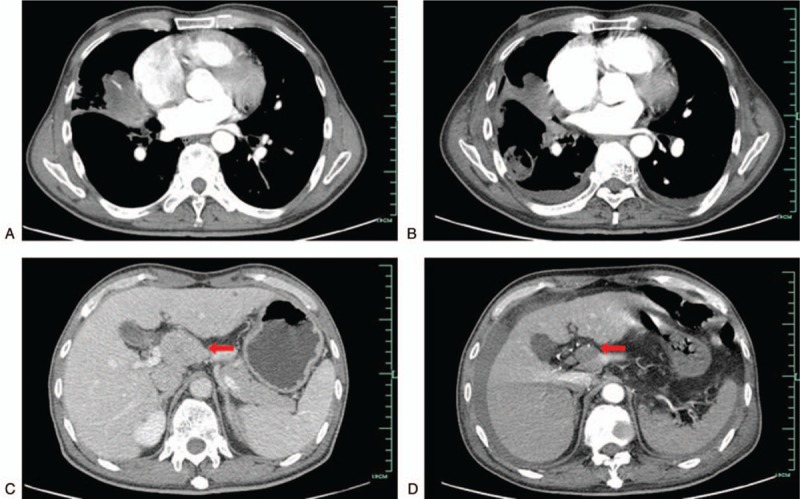
Computed tomograhy presented the changes of lesions before and after Apatinib treatment. A/C: primary pulmonary inflammatory myofibroblastic tumor (IMT) (A) and intra-abdominal metastasis (C) before Apatinib treatment. B/D: primary pulmonary IMT (B) and intra-abdominal metastasis (D) after Apatinib treatment for 5 months.

In February 2018, 3 months after the lung biopsy, the patient complained about cough and lumbago. The chest CT manifested that the mass in the right middle lobe of the lung enlarged to 6.2 cm × 3.8 cm (Fig. 3A), and new diffused nodules appeared. At the same time, thoracic vertebra metastasis was also indicated by the chest CT (Fig. 1C). Then multiple bone metastases were proved by an emission CT for bones. An intra-abdominal mass near porta hepatis that indicated metastasis was showed in further the abdominal CT and MRI (Fig. 3C). So the nature of the tumor was suspectable. Subsequently, the re-biopsy of the lesion in the right lung was conducted, and the pathological result was totally consistent to the previous one. This case was discussed in a multidiscipline meeting, during which pathologists still inclined to the diagnosis of IMT. The histopathologic diagnosis was confirmed in another center. Afterwards, the patient underwent a bone marrow biopsy. The histopathologic result indicated that bone marrow was infiltrated by spindle cells which were similar to the tumor cells in the previous samples from the lung biopsy, morphologically and immunohistochemically (Fig. 2D). Other distant metastases were excluded by work-up examinations, including the pelvic MRI, gastroscopy, colonoscopy, and the brain MRI. Besides, endoscopic views of erosive gastritis with multiple ulcers were observed. Even so, in consideration of the rapid progress, the members of the multidisciplinary team reached a consensus on the therapy with Celecoxib, a selective cyclooxygenase-2 (COX-2) inhibitor. Therefore, the patient orally took Celecoxib at the dose of 0.2 g twice a day from February 2018. However, he stopped taking the medicine because of the aggravated gastric ulcer 1 month later, although it could be relieved by acid inhibitors. Nevertheless, there was no remarkable improvement after the drug withdrawal according to the chest CT scan. Based on the outcome of multidisciplinary meeting, Apatinib was administrated at the dose of 250 mg once a day from March 2018 to now.

On the follow-up in August 2018, the mass of abdominal cavity significantly shrank with the resulting partial remission (PR) obtained by an abdominal CT scan according to the Response Evaluation Criteria in Solid Tumors (RECIST) Version 1.1 standard (Fig. 3B). The previous lung lesions also decreased in size by chest CT and was defined as a stable disease (SD) (Fig. 3D), whereas new lesions with cavity appeared. The lung biopsy of one new nodule revealed that the lesion was a focus of inflammation and the antibiotic therapy was effective. Fungal infection was also excluded by the further tissue culture. When receiving Apatinib, the patient presented hypertension and hand-foot syndrome, both of grade 1 severity according to the National Cancer Institute Common Toxicity Criteria for adverse events version 4.0. His last follow-up was in December 2018 when he was in good condition, and the masses of lung and abdominal cavity kept stable compared with the condition in August 2018.

## 3 Discussion

Originally, IMT is usually considered as an inflammatory or reactive lesion, resulting in a benign clinical course. Although there are debates over the pathogenesis of IMT, infection, trauma, and autoimmune diseases are commonly proposed as the causes.^[5]^ In this study, the patient has suffered from recurrent respiratory tract infection, which suggests that infection may be the key factor contributing to the pathogenesis of IMT. However, current clinical data and molecular analysis suggest that IMT is a true neoplasm and present an intermediate biological behavior with a tendency to recurrent, malignant transformation and metastasis. The discovery of clonally rearrangement of ALK gene provides essential evidence for the neoplastic property of IMT rather than an inflammatory lesion.^[6]^ Just as our report indicates, IMT has the possibility to be morphologically benign and ALK-negative, but behaves aggressively. However, there is no reasonable explanation, and further investigations are in need.

Regarding to metastatic IMT, only sporadic cases have been reported in the previous studies. The most cases are abdominal IMT and retroperitoneal IMT with regional metastasis.^[7]^ Nevertheless, unusual cases have also been reported, such as a primary breast IMT metastasizing to the left groin area after surgery^[8]^ and a thyroid IMT with metastasis to the right adductor Magnus.^[9]^ Distant metastasis from a pulmonary IMT is extremely rare. To the best of our knowledge, there have been only 2 related case reports. One described a middle-aged patient with pulmonary IMT who underwent a complete resection. Two years later, multiple bone recurrences were observed by a bone CT scan. Then spinal cord compression and metastasis to intra-abdominal organs were diagnosed. Finally, pleural effusion, liver, adrenal, and spleen metastases were revealed by the CT scan.^[10]^ The other case was about an old female who had a history of pneumonia and was surgically treated. She complained about swelling and pain in the left upper maxillary region 2 years after pulmonary resection. An infiltrative lesion in the left maxillary sinus was showed by CT, with destruction of the maxillary bone. A maxillary IMT was identified pathologically after the surgery. As histopathological results of the previous lung lesions were reviewed, the diagnosis of pulmonary IMT with metastasis to the maxillary region was confirmed.^[11]^ Our distinctive case presents the metastases to bone, abdominal cavity, and bone marrow, which adds another scarce example to the list of multimetastatic pulmonary IMT cases.

The clinical features of pulmonary IMT are usually unspecific, which always makes the diagnosis difficult to carry out. IMT is asymptomatic in many patients, whereas in others respiratory tract infections related symptoms, such as cough, short of breath, chest pain or complaints of fatigue, fever and weight loss, commonly appear. Laboratory examinations in most instances are normal, but sometimes anemia, thrombocytopenia, and an elevated sedimentation rate may be revealed. Radiographically, a pulmonary IMT usually occurs as a single nodule or a mass with clear boundary and slight to moderate homogeneous contrast enhancement, but occasionally it may mimic malignant tumors. According to reports, the incidence of multiple nodules accounts for 5% of the pulmonary IMT.^[12]^ The current case manifests 2 synchronous masses located in bilateral lung, which is another unique feature. It is reported that the accurate pathological diagnosis of IMT depends on surgical specimen that means needle biopsy cannot be considered as a reliable diagnostic method. But for inoperable patients, needle biopsy should be reckoned as an appropriate way to obtain pathological information. In this report, the patient underwent biopsy procedures for 4 times, and 3 of them revealed spindle cells, showing significant diagnostic values. Pathological results of the first 2 CT-guided needle biopsies of lung lesions consistently suggested the diagnosis of IMT. The bone marrow biopsy confirmed this diagnosis and, more precisely, demonstrated an unusual metastatic site of this entity. As far as we know, it has been the first case report with bone marrow metastasis involved in a pulmonary IMT.

Synchronous lesions of different organs are exactly present in a few cases of IMT.^[13]^ In this case, we confirm the diagnosis of metastatic spread rather than multifocal lesions. The reasons are as follows: first, all the lesions of bone, abdominal cavity, and bone marrow infiltration have appeared subsequently to the lung occupation; second, the pulmonary IMT with bone marrow infiltration by spinal cells has been confirmed by pathological examinations; finally, although IMT with bone marrow metastasis has never been reported, a few examples of solid-malignant-tumor like prostate cancer, breast cancer, small cell lung cancer, neuroblastoma, and gastric cancer can occasionally metastasize to bone marrow despite of the low incidence. In addition, as a small minority of IMT can metastasize owing to malignant transformation,^[14]^ we have ruled out this possibility by the examination of the pathological samples from the re-biopsy of the lung lesion and a bone marrow biopsy. Malignant pathological features including cellular atypia and a high mitotic rate especially atypical mitosis of the tumor cells have not been observed. Therefore, the diagnosis of the pulmonary IMT with multiple metastases of this patient is reliable.

In general, IMT patients who are feasible for surgical section present a favorable prognosis with 5- and 10-year survival of 91% and 77%, respectively.^[15]^ Nevertheless, it is reported that approximately 8% of pulmonary IMT without indications for surgery shows a continuous growth with a proportion of 5% in risk of distant metastasis.^[16]^ With widely varied prognosis, IMT has no definite predictor for its recurrence or metastasis. ALK rearrangement is a significant characteristic of IMT. Relationship between the clinical outcomes of IMT and ALK expression remains controversial. Previous studies indicate that the expression of ALK relates to better clinical outcomes. ALK-rearranged patients are more likely to suffer from local recurrence, but metastasis is confined to ALK-negative patients.[^4^,^17^] Extra evidence of our case is now added to this conclusion. However, a clinical retrospective report shows no statistically significant correlation among ALK-expression, tumor type, recurrence, and metastasis.^[18]^

Owing to the rarity and curative effect of operation, clinical data over the treatment of inoperable IMT is limited, which mainly can be obtained from small-scale respective clinical studies and case reports. To date, there has been no standard treatment for inoperable IMT cases. It is reported that surgery is the best treatment for recurrent pulmonary IMT when patients are eligible.^[19]^ Crizotinib, an ALK inhibitor, is sensitive to ALK-expression IMT.^[20]^ Recently, a phase 1b open-label study including 9 ALK-positive IMT cases has revealed that the objective response rate is 67%, and the progression-free survival at 2 years is 66.7%.^[21]^ Nevertheless, as for ALK-negative inoperable patients including cases of unresectable recurrence or metastasis, therapeutic approaches, such as steroids, NSAIDs, chemotherapies, and radiotherapy, have only exhibited unsatisfactory effects. Steroids or NSAIDs may be clinically beneficial to inoperable IMT patients, but the majority of related studies simply refer to extrapulmonary tumors of children.[^18^,^22^,^23^,^24^] There has been only 1 case report involving NSAID monotherapy on adult IMT of lung origin, in which case Celecoxib brings about a complete remission.^[25]^ Besides, chemotherapy or radiotherapy for IMT patients has shown contradictory results. As a report goes, chemotherapies like the combination of epirubicin, dacarbazine, and docetaxel or vinorelbine plus methotrexate may be viable therapeutic options for IMT.[^26^,^27^] However, the efficacy has not been verified in the cases of pulmonary IMT. Thus, given the current difficulties in the management of inoperable IMT, new attempts are requisite. Tricia et al explored the expression of Programmed Cell Death-Ligand 1 (PD-L1) in 28 IMT cases, unveiling that PD-L1 expression occurred in 80% of recurrent and metastatic tumors and 88% of ALK-negative tumors, providing a rationale for further investigations on the utility of checkpoint blockade therapy in the treatment of refractory IMT.^[28]^

Apatinib is an oral small-molecule tyrosine kinase inhibitor of VEGFR-2. Clinical trials have proved that Apatinib is effective for patients with chemotherapy-refractory gastric cancer, advanced non-small-cell lung cancer expressing wild-type epidermal growth factor receptor, breast cancer, and hepatocellular carcinoma.^[29]^ Recently, Apatinib has been reported to show excellent effects on advanced sarcoma,^[30]^ ovarian cancer^[31]^ and thyroid cancer.^[32]^ Despite the significant effects on various solid tumors, treatments with Apatinib or other anti-angiogenesis drugs on IMT have never been reported previously. Our case demonstrates that Apatinib is also efficacious on advanced IMT, featuring significant shrinking of the intra-abdominal lesion and slight decrease in size of the lung lesions after a 5-month administration.

## 4 Conclusion

In conclusion, pulmonary IMT shows the potential for metastasis, even to multiple anatomic locations. Bone marrow may be an unusual metastatic site. In clinical practices, work-up examinations including bone marrow biopsy are necessary to exclude distant metastasis before treatment. When a systematic therapy fails, Apatinib can be taken into consideration for the treatment of inoperable pulmonary IMT patients who lack ALK rearrangement.

## Acknowledgment

The authors thank the financial support from Medical Scientific Research Foundation of Zhejiang Province of China (no: 2018KY832) and Science Foundation of Shaoxing People's Hospital for Young Scientists (no: 2017B02).

## Author contributions

**Conceptualization:** Qiuxia Liu, Jianfang Wang.

**Data curation:** Qiuxia Liu, Jianguo Wei, Xizhong Liu, Jianfang Wang.

**Investigation:** Qiuxia Liu, Jianguo Wei, Xizhong Liu, Jianfang Wang.

**Resources:** Qiuxia Liu, Jianguo Wei, Xizhong Liu, Jianfang Wang.

**Supervision:** Qiuxia Liu, Jianfang Wang.

**Writing – original draft:** Qiuxia Liu.
